# Is Vascular Amyloidosis Intertwined with Arterial Aging, Hypertension and Atherosclerosis?

**DOI:** 10.3389/fgene.2017.00126

**Published:** 2017-10-06

**Authors:** Yushi Wang, Xiaoxing Feng, Botao Shen, Jing Ma, Waiou Zhao

**Affiliations:** Cardiovascular Center, The First Hospital of Jilin University, Changchun, China

**Keywords:** vascular amyloidosis, intertwine, vascular aging, hypertension, atherosclerosis

## Abstract

Vascular amyloidosis (VA) is a component of aging, but both VA and aging move forward together. Although, not all age-related molecules are involved with VA, some molecules are involved in a crosstalk between both of them. However, the cellular mechanism by which, vascular cells are phenotypically shifted to arterial remodeling, is not only involved in aging but also linked to VA. Additionally, patients with hypertension and atherosclerosis are susceptible to VA, while amyloidosis alone may provide fertile soil for the initiation and progression of subsequent hypertension and atherosclerosis. It is known that hypertension, atherosclerosis and amyloidosis can be viewed as accelerated aging. This review summarizes the available experimental and clinical evidence to help the reader to understand the advance and underlying mechanisms for VA involvement in and interaction with aging. Taken together, it is clear that VA, hypertension and atherosclerosis are closely intertwined with arterial aging as equal partners.

## Introduction

Amyloid is found in the aortic walls of almost 100% of the population above 50 years of age (Mucchiano et al., 1992), and also aged people are susceptible to hypertension and atherosclerosis, which indicates that vascular amyloidosis (VA), hypertension and atherosclerosis are highly associated with aging. However, few studies have focused on the relationship between amyloidosis and arterial diseases. We reviewed the molecular and cellular mechanisms of VA and give evidence how strongly it is fundamentally intertwined together with hypertension, atherosclerosis, and aging at the cellular and molecular levels (Wang et al., 2010a).

## Distribution of amyloid proteins

Amyloidosis is a disorder of protein metabolism characterized by extracellular accumulation of abnormal insoluble amyloid fibrils. About 30 proteins are known to form pathogenic amyloid or amyloid-like fibrillary networks in a wide range of human tissues which are associated with diseases having high morbidity and mortality rates (Galant et al., 2017). However, there are only four kinds of amyloid proteins which are mainly associated with VA. Here we review the four main amyloid proteins that are susceptible to deposit in different arteries and interact with different cells (Table 1).

**Table 1:** The relationships among the four kinds of amyloid proteins, the arteries likely deposited, the cells likely involved and references.

**Amyloid protein**	**Arterial tree**	**Cellular role**
TTR	Coronary Booth et al., 1995, Cerebral Sekijima, 2015	EC Nunes et al., 2013
Apo1	Coronary Stewart et al., 2007, Aorta Mucchiano et al., 2001	Matrix Ramella et al., 2011
Immunoglobin γ	Cerebra Audemard et al., 2012	EC Truran et al., 2014, Matrix Berghoff et al., 2003
Medin	Aorta Davies et al., 2015a, Cerebral Peng et al., 2005	EC Davies et al., 2015b, VSMC Hagggvist et al., 1999

In general, these four amyloid proteins TTR (Transthyretin), Apo1 (Apolipoprotein A-1), immunoglobin γ, and medin are susceptible to deposit, respectively at cerebral artery, coronary artery and aorta. If amyloid proteins deposit within the walls of the cerebral vasculature with subsequent aggressive vascular inflammation, it will lead to recurrent hemorrhagic strokes (Agyare et al., 2014); If they deposit within the walls of the coronary artery, they will lead to angina pectoris, even ischemia cardiomyopathy; If they deposit within the wall of aorta, they will lead to hypertension, atherosclerosis, and even dissecting aneurysm eventually (Wang et al., 2010a).

## Hypertension and VA

What's the relationship between VA and hypertension? Let's review it. In 1997, Rotterdam Study showed that hypertension, as an indicator of atherosclerosis, was not only related to vascular dementia, but also Alzheimer's disease (AD) (Forette et al., 1998). Hypertension may initiate vascular damage before the onset of AD so as to make symptoms more pronounced and progress more rapidly (Liu et al., 2014). Furthermore, compromised vessels may be more vulnerable to the deleterious effects of amyloid β-protein (Aβ). For example, since blood brain barrier (BBB) integrity is damaged by hypertension, blood components may enter the brain prior to the large-scale accumulation of Aβ. These blood components may serve as a seed for Aβ deposition and promote vascular inflammation, resulting in cellular damage and the release of toxic molecules. It is suggested that hypertension has a significant effect on the onset and progression of AD. Epidemiological studies have shown that AD rapidly progressed in elderly hypertensive people. It is also suggested by numerous epidemiological studies that treating hypertension in midlife may be an effective strategy for reducing the likelihood of AD onset later in life (Kruyer et al., 2015).

Conversely, VA, as a main part of vascular aging, is involved with age-associated vascular remodeling. During the whole process, every step is linked closely to the initiation and progression of hypertension. Endothelial dysfunction leads to hypertension directly with the decrease of nitric oxide since endothelium-dependent vasodilation is impaired (Puddu et al., 2000). VSMCs, as the most important cells of vascular wall, shift the phenotypes during VA, which eventually lead to increase vascular wall stiffening, decrease elasticity of vascular wall and hypertension occurs subsequently (Wang et al., 2014a). Fibroblasts, as the main cells in the adventitia of vascular wall, once activated during amyloidosis, affect both intima and adventitia thickening, which make hypertension accompany with it (Peng et al., 2002). The vascular extracellular matrix (ECM) also is involved in amyloidosis and hypertension due to the deposition of amyloid proteins in the ECM, and arterial elasticity can be injured so that hypertension ensues (Larsson et al., 2006). Collectively, all hypertensive patients are vulnerable to VA (Kruyer et al., 2015), while VA accelerates the initiation and progression of hypertension (Larsson et al., 2006).

## Atherosclerosis and VA

Similarly, it is certain that the relation between atherosclerosis and VA exists. Roher et al. (2003) found that cerebrovascular atherosclerosis is significantly more frequent and severe in those with AD compared with normal aging and those with other neurodegenerative diseases. Also, Beach et al. (2007) found there was an association between circle of Willis atherosclerosis and AD. Furthermore, they found that this association is relatively specific to AD rather than other major neurodegenerative diseases. Alternatively, the finding that amyloid-β is present in advanced human atherosclerotic lesions suggests that atherosclerosis may be promoted directly by AD pathology, potentially through altering the metabolism of oxidized lipoproteins (Yarchoan et al., 2012). Moreover, the micro-environment produced by MFG-E8 and medin who are intimately involved with VA through promoting the secretion of many inflammatory molecules and the phenotypic shifts of vascular cells render the arterial wall a fertile soil for atherosclerosis to flourish.

## Molecular mechanisms of vascular amyloidosis

**Figure 1:**
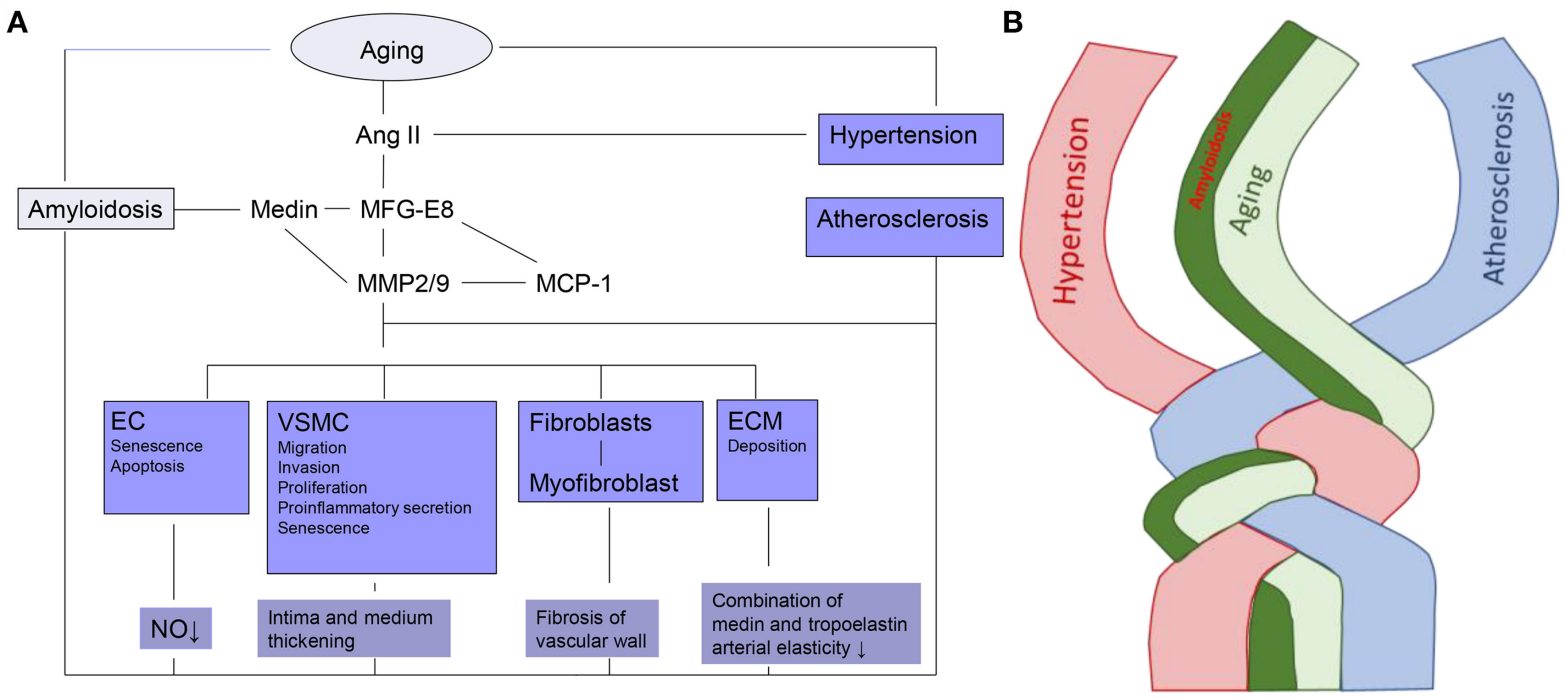
The linkage and twist of VA with aging. **(A)** Cellular and molecular mechanisms of VA. Ec, endothelial cell; VSMC, vascular smooth muscle cell; ECM, extracellular matrix; Ang II, Angiotensin II; MFG-E8, Milk fat globule-epidermal growth factor-8; MMP2/9-zinc, containing endopeptidases2/9; MCP-1, Monocyte chemoattractant protein-1; NO, Nitric oxide. **(B)** Amyloidosis is intertwined with arterial aging, hypertension and atherosclerosis.

It has been demonstrated that MFG-E8 and medin are rich in the “amyloid-like” aortic media. Simultaneously, MFG-E8, as a novel marker of vascular aging, induces proinflammatory molecules which are involved within the Ang II signal pathway, including Medin, MMP-2/9, MCP-1, as outlined in Figure 1.

### MFG-E8

Milk fat globule-epidermal growth factor-8 (MFG-E8), is also known as lactadherin. Growing evidence has indicated that MFG-E8 is a secreted inflammatory mediator that orchestrates diverse cellular interactions involved in the pathogenesis of various diseases, including vascular aging and amyloidosis. During aging, as the downstream molecule of Ang II signal pathway, both MFG-E8 transcription and translation increase within the arterial walls of various species (Fu et al., 2009). Moreover, many downstream inflammatory molecules within the Ang II signal pathway are induced by MFG-E8. During amyloidosis, as the origin of amyloid protein, MFG-E8 cleaves into medin which increases the stiffness of vascular wall through the binding to tropoelastin. Furthermore, MFG-E8 leads to the phenotypic shifts of all kinds of vascular cells which supply a fertile soil for arterial diseases. Therefore, MFG-E8 makes VA, aging, hypertension and atherosclerosis intertwine together.

### Medin

Vascular amyloidosis (VA) is markedly increased with aging (Wang et al., 2013). As mentioned, medin is the most common amyloid protein which deposits in the wall of aorta. Prior studies have shown that a 50 amino acid polypepetide called medin, cleaved from MFG-E8's C2-like domain (Hagggvist et al., 1999), not only tightly binds to tropoelastin but also eventually incorporates into arterial amyloids (Larsson et al., 2006). These medin amyloids have been observed within arterial walls, including that of both aorta and temporal artery (Peng et al., 2005), in Caucasian populations over 50 years old (Davies et al., 2014, 2015b). Recent studies have shown that aortic medin amyloids serve as a trigger for amyloid A-derived amyloidosis (Larsson et al., 2011).

### Ang II

Ang II, as a leading proinflammatory vasoactive stressor, can induce the inflammatory molecules in the signaling cascade within the aged arterial wall, in which VA may be progressing. Augmentation of these Ang II biosignals appear to be the foundation of the molecular mechanisms of age-associated adverse arterial structural remodeling (Wang et al., 2010b). It is known that “local” Ang II regulated independently is over 1,000-fold more abundant than “circulating” Ang II and plays an important role in vascular pathophysiology in elderly people (Diz, 2008). Some studies have shown that expression of Ang II protein increases not only in the aged aortic wall of rats but also in the thickened intima of older monkey aortae (Wang et al., 2003; Jiang et al., 2008). Moreover, the immunofluorescence of Ang II increases within the “grossly normal” aortic wall of older people (Wang et al., 2007). Simultaneously, it has been found that MFG-E8 can be induced by Ang II in VSMCs isolated from rat aortae, which indicates that MFG-E8 is required for Ang II to increase some downstream inflammatory molecules. Consequently, MFG-E8, which plays a crucial role in arterial aging and amyloidosis, is a novel link between them.

### MMP-2/9

The zinc-containing endopeptidases, MMP-2/-9, are involved in the breakdown of ECM occurred in amyloidosis, aging, hypertension and atherosclerosis (Wang et al., 2014b, 2015). The activity of MMP-2/9 increases with amyloidosis (Peng et al., 2007) and advancing age within the aortic wall which is linked to increased Ang II/MFG-E8/medin signaling (Wang et al., 2003, 2005, 2007). It has been shown that MMP-2/9 activity *in situ* is enhanced both in the “grossly normal” aortic segments of old people (Wang et al., 2010b) and in thoracic aortic aneurysms and dissections (Peng et al., 2007). The proinflammatory microenvironment MMPs create through modifying ECM and the introduction of inflammatory molecules shifts the phenotypes of vascular cells, including endothelial cells (ECs), vascular smooth muscle cells (VSMCs), and fibroblasts, which are so-called arterial remodeling occurred in various arterial diseases. Therefore, MMP2/9, is another group of key factors that intertwines amyloidosis, aging, hypertension and atherosclerosis.

### MCP-1

MCP-1, which belongs to the G-protein coupled receptor 1 family, is a notorious inflammatory cytokine downstream of Ang II signaling in the cardiovascular system (Wang and Shah, 2015) and originally functions by recruiting immune cells to sites of inflammation. Vascular amyloidosis (VA), aging, hypertension and atherosclerosis are all a chronic, low-grade inflammation (Wang et al., 2014b, 2015), therefore, MCP-1 is involved intimately in the inflammatory process of arterial diseases. Additionally, it has been demonstrated that MFG-E8 induced by Ang II promotes the expression of MCP-1 in VSMCs within the old rat aortic wall, which leads MCP-1 within the Ang II/MFG-E8/VSMC invasion signaling cascade (Wang et al., 2014a). The relationship between MCP-1 and MFG-E8 can be viewed as an important signal relationship in both VA and aging.

## Cellular mechanisms of amyloidosis

In arterial diseases, such as aging, amyloidosis, hypertension, and atherosclerosis, all the arterial cells including ECs, VSMC, fibroblasts, and matrix are ultimately the downstream targets of the signal molecules. Adverse arterial restructuring which occurs in all arterial diseases is the result of phenotypic shifts of those arterial cells. The amyloidosis-related molecule MFG-E8 and medin have already been proven to have a close relationship with the arterial cells and matrix, as illustrated in Figure 1.

### Endothelial cells (ECs)

Endothelial integrity is important to vascular health, with ECs building the frontline cells of the arterial wall (Wang et al., 2014b). It is suggested that the amyloidosis associated protein medin is toxic to aortic ECs *in vitro* (Madine and Middleton, 2010) and may underlie the pathogenesis of aortic aneurysm *in vivo* through a weakening of the aortic wall (Peng et al., 2007). In addition, the increased inflammatory load, such as elevated MFG-E8 in the old endothelia may damage endothelial mitochondrial DNA and interfere with the mitochondria life cycle via enhanced ROS generation, which consequently initiates and promotes EC senescence and apoptosis (Wang et al., 2005, 2007). These cellular events and micro-environments lead to endothelia dysfunction which renders the arterial wall a fertile soil in which amyloidosis and atherosclerosis may flourish (Najjar et al., 2005; Wang et al., 2014b). Interestingly, endothelial dysfunction also occurs with aging even in healthy adults (Sepulveda et al., 2017). additionally, due to decreased bioavailability of nitric oxide, endothelial dysfunction which impairs endothelium-dependent vasodilation in hypertension (Puddu et al., 2000) may precede the development of clinical hypertension (Najjar et al., 2005). Collectively, endothelial dysfunction can be viewed as a prelude for arterial disease.

### Vascular smooth muscle cells (VSMCs)

The phenotypic shifts of VSMC, including enhanced migration, invasion, proliferation, proinflammatory secretion, and senescence, are the most important characters of vascular aging and amyloidosis. They also supply a fertile stage for the initiation and progression of the pathogenesis of hypertension and atherosclerosis in the elderly (Wang et al., 2014a,b). However, those phenotypic shifts are associated directly with the inflammatory molecules Ang II, MFG-E8, and MCP-1. Young VSMCs with the treatment of Ang II secrete MFG-E8 to levels similar to old untreated cells (Gao et al., 2008; Fu et al., 2009). MFG-E8 is induced by Ang II in aging, while MFG-E8 in amyloidosis induces the expression of MCP-1 in VSMCs within the rat aortic wall (Fu et al., 2009), leading to invasion of VSMC. Additionally, it is well-known that MFG-E8, the precursor of medin, is abundantly expressed by VSMCs (Hagggvist et al., 1999; Peng et al., 2002, 2005). Moreover, it has been shown that aortic amyloidosis is also involved with a proinflammatory VSMC phenotypic shift due to the accumulation of MFG-E8 and medin in the aorta (Wang et al., 2014b). Chronic exposure of VSMC to intact MFG-E8 markedly increases proliferation and invasion (Fu et al., 2009; Wang et al., 2012), while it is shown that chronic exposure of VSMC to medin fragments significantly increase secreted MMP-2 levels, which promote phenotypic shifts of vascular cells through the proinflammatory microenvironment (Peng et al., 2007; Lakatta, 2013).

### Fibroblasts

Fibroblasts compose the major vascular cells in the adventitia which serves as the artery's final line of defense (Michel et al., 2007). A growing body of evidence indicates that adventitial remodeling intertwines with the VSMC phenotypic shift (Sierevogel et al., 2002; Moos et al., 2005; Passman et al., 2008; Grabner et al., 2009). Variations in the adventitia could be a signal of impending vascular disorders (Wang et al., 2010b). With advancing age or amyloidosis, fibroblasts become activated, synthesize smooth muscle actin and transit into myofibroblasts which contribute to both intimal and adventitial thickening (Najjar et al., 2005; Sepulveda et al., 2017). Additionally, the adventitia and adjacent perivascular white adipose tissue, similar to the intima, is a major source of MMPs (Najjar et al., 2005; Gao et al., 2008; Wang et al., 2012; Sepulveda et al., 2017). The transition of fibroblasts to myofibroblasts points to the remodeling of adventitia and the activation of MMPs (Sierevogel et al., 2002). Therefore, the inhibition of the activation of MMPs may help to retard the progress of arterial diseases.

### ECM

The ECM is not only essential for the structural integrity of arterial walls but also serves as a platform for the binding of vascular cell molecules that are crucial for intercellular communication (Wang et al., 2013). Immunostaining and proteomics analyses have also demonstrated that MFG-E8 is a secreted extracellular adhesive molecule within the aortic extracellular space (Hagggvist et al., 1999), while medin, as a unique amyloid protein, is deposited exactly in the ECM (Wang et al., 2013). Furthermore, the MFG-E8-derived medin is not only co-localized with elastic fibers of older arteries in the ECM but also binds to tropo-elastin in a concentration-dependent fashion, forming amyloid-like fibrils within the extracellular space of arterial walls (Larsson et al., 2006; Lakatta, 2013). The related arteries can't stretch and recoil freely as they are subjected to decreased arterial elasticity (Larsson et al., 2006), which leads to hypertension through arterial stiffening which is a hallmark of aging (Zieman et al., 2005). From this view point, amyloidosis is closely intertwined with aging and hypertension.

## Functional variations in amyloidosis tissues

It is known that about 80% of Alzheimer's disease (AD) patients show some degree of Cerebral amyloid angiopathy (CAA) (Michel et al., 2007). CAA is characterized by the deposition of amyloid beta (Aβ) proteins within the walls of small to medium-sized blood vessels of the brain (Agyare et al., 2014). It has been demonstrated that AD patients with CAA manifest a declining cognitive test performance compared to their early life time. It is believed that several mechanisms linking CAA to cognition include cerebrovascular inflammation, vascular dysfunction (Viswanathan et al., 2008; Arvanitakis et al., 2011; Urbach, 2011; Agyare et al., 2014), oxidative stress, tissue microstructural changes, etc. Additional studies are needed to help elucidate the role of these factors in the relationship between CAA and cognition decline (Greenberg, 2002; Arvanitakis et al., 2011).

If coronary blood flow decreases due to the deposition of amyloid, it will lead to angina pectoris, or even ischemia cardiomyopathy (Bulut et al., 2016). It has been suggested that several mechanisms are involved in the coronary microvascular dysfunction, including wall thickening, luminal narrowing of the vessels due to intramural amyloid deposition in the vessel wall, perivascular and interstitial amyloid deposits causing extrinsic compression of the microvasculature, and endothelial and autonomic dysfunction (Kawata et al., 2013; Bulut et al., 2016).

If amyloid protein, especially medin, deposits within the wall of the aorta, it will lead to hypertension, atherosclerosis and dissecting aneurysm. As we mentioned earlier, it is found that medin has a close association with the internal elastic lamina, which is not only involved with stiffening of aortic wall but also underlies the pathogenesis of sporadic thoracic aortic aneurysm via weakening of the aortic wall (Madine and Middleton, 2010). Annika Larsson, etc. found that aortae from patients suffering from thoracic aortic aneurysm and dissection contain higher levels of medin than normal aortae (Larsson et al., 2007). Westermark and colleagues proved that widely spread medin amyloid deposits in the thoracic aorta are associated with aortic wall weakening. It is notable that other forms of amyloidosis involving arteries can lead to an increased risk for rupture (Peng et al., 2007). In addition, VSMCs incubated together with medin increase the production of matrix metalloproteinase-2 which degrades elastin and collagen and eventually weakens the vessel wall (Peng et al., 2007).

## Conclusions

Collectively, the prevalence of amyloid diseases in the aging population has become a major health concern and consequently many resources are being invested into developing therapies (Merlini and Westermark, 2004). Indeed, it appears that VA may be strongly intertwined with arterial aging, hypertension and atherosclerosis. Since they all have a close relationship with inflammation, targeting proinflammatory signaling molecules, including Ang II, MFG-E8, medin, and MMPs, etc. would be promising approaches to prevent the pathogenesis of clinical cardiovascular events.

## Author contributions

YW: Writing the paper. XF: Research of literatures. BS: Research of literatures. JM: Final editing. WZ: Concept of the review.

### Conflict of interest statement

The authors declare that the research was conducted in the absence of any commercial or financial relationships that could be construed as a potential conflict of interest.
